# Adrenal cortex senescence: an ageing-related pathology?

**DOI:** 10.1007/s40618-025-02566-9

**Published:** 2025-03-25

**Authors:** Emma Short, Ramzi Ajjan, Thomas M. Barber, Ian Benson, Victoria Higginbotham, Robert Huckstepp, Venkateswarlu Kanamarlapudi, Natasha Mumwiro, Stuart R. G. Calimport, Barry Bentley

**Affiliations:** 1 Cardiff School of Technologies, Cardiff Metropolitan University, Cardiff, UK; 2 Department of Cellular Pathology, Swansea Bay University Health Board, Swansea, UK; 3 Leeds Institute of Cardiovascular and Metabolic Medicine, University of Leeds, Leeds, UK; 4 Warwickshire Institute for the Study of Diabetes, Endocrinology and Metabolism, University Hospitals Coventry and Warwickshire, Clifford Bridge Road, Coventry, UK; 5 Division of Biomedical Sciences, Warwick Medical School, University of Warwick, Coventry, UK; 6 University of Glasgow Medical School, Glasgow, UK; 7 Swansea University Medical School, Swansea University, Swansea, UK; 8 School of Life Sciences, University of Warwick, Coventry, UK; 9 University of Birmingham Medical School, Birmingham, UK; 10 Collaboration for the Advancement of Sustainable Medical Innovation (CASMI), University College London, London, UK; 11 Center for Engineering in Medicine and Surgery, Harvard Medical School, Boston, MA USA; 12 Department of Surgery, Massachusetts General Hospital, Harvard Medical School, Boston, MA USA; 13 Shriners Children’s, Boston, MA USA

**Keywords:** Adrenal cortex senescence, Adrenal cortex ageing, Senescence, Healthy longevity

## Abstract

The adrenal glands are a pair of endocrine organs that produce and secrete mineralocorticoids, glucocorticoids, sex hormones, adrenaline, and noradrenaline. They have a vital role in a range of physiological processes including regulating electrolyte balance, blood pressure and metabolism, immunomodulation, sexual development and the stress response. Adrenal cortex senescence describes the ageing-related decline in the normal functioning of the adrenal cortex, characterised by an alteration in the output of adrenal cortical hormones, in particular reduced secretion of dehydroepiandrosterone (DHEA) and sulfated dehydroepiandrosterone (DHEAS). Such endocrine aberrations may be implicated in adverse clinical outcomes including mood disturbances, impairment in cognitive functioning, metabolic dysfunction and osteopenia. This paper shall address whether adrenal cortex senescence should be recognised as an ageing-related pathology, which has recently been defined as one that develops and/or progresses with increasing chronological age, that is associated with, or contributes to, functional decline, and is evidenced by studies in humans.

## Introduction

**Fig. 1:**
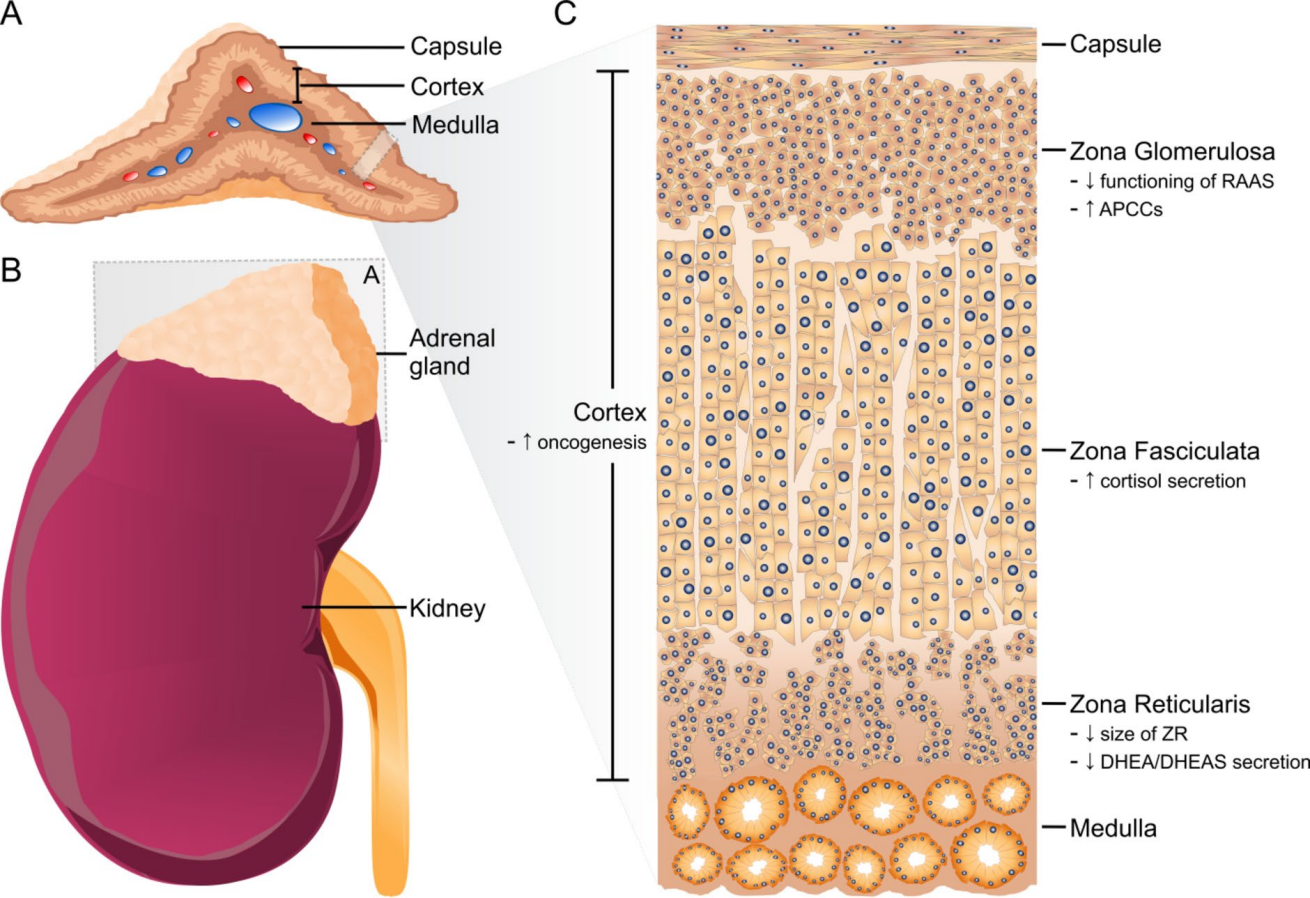
**A** The adrenal glands are encapsulated organs comprising a cortex and medulla. They are located above the upper pole of the kidneys (**B**). **C** The adrenal cortex is composed of the zona glomerulosa (ZG), zona fasciculata (ZF) and zona reticularis (ZR). The key ageing-related changes of the adrenal cortex that have been reported in human studies are highlighted in (**C)**: There is suboptimal functioning of the renin-angiotensin-aldosterone system (RAAS), there are increased aldosterone-producing cell clusters (APCC), there is increased secretion of cortisol, there is a reduction in the size of the zona reticularis, there is reduced secretion of DHEA/ DHEAS and there is an increased prevalence of adrenal tumours

The adrenal glands are a pair of retroperitoneal endocrine organs that are located above the upper pole of the kidneys. Each gland measures approximately 5 × 2 × 1 cm and weighs up to 5 g [1]. The glands are composed of two distinct zones, the outer cortex, of mesodermal origin, and the inner medulla, derived from neuroectoderm (Fig. 1). The cortex comprises [1]:

- the zona glomerulosa (ZG), responsible for the production of mineralocorticoids, primarily aldosterone.
- the zona fasciculata (ZF), producing glucocorticoids, of key importance is cortisol.
- the zona reticularis (ZR), producing androgens, primarily dehydroepiandrosterone (DHEA), which can be sulfated to dehydroepiandrosterone-sulfate (DHEAS).

The adrenal medulla synthesises the catecholamines noradrenaline and adrenaline [1].

The functions of the adrenal glands are related to their hormonal output, and include regulating electrolyte balance, blood pressure and metabolism, immunomodulation, sexual development and the stress response [1, 2].

The adrenal cortex undergoes a range of ageing-related structural and functional changes. In 2019, Calimport et al. called for the systematic and comprehensive classification of ageing-related pathologies at the metabolic, tissue, organ and systemic levels following the World Health Organisation’s decision to officially classify ageing-related aetiology within the International Classification of Diseases (ICD-11) [3, 4]. To address this, the International Consortium for the Classification of Ageing-Related Pathologies (ICCARP) was established in 2023, led by Cardiff Metropolitan University [5]. The ICCARP has recently defined the criteria for an ageing-related pathology as one that [5]:

1. Develops and/or progresses with increasing chronological age;
2. Should be associated with, or contribute to, functional decline, or an increased susceptibility to functional decline;
3. Is evidenced by studies in humans.

Here we review the evidence to determine whether adrenal cortex senescence should be recognised and classified as an ageing-related pathology.

## Adrenal cortex senescence

We, the ICCARP Endocrine and Metabolic working group, hypothesise that adrenal cortex senescence might be an ageing-related pathology, as it describes an ageing-related decline in the normal functioning of the adrenal gland, characterised by an alteration in the output of adrenal cortical hormones, in particular a reduction in the secretion of DHEA and DHEAS. These endocrine aberrations are associated with adverse clinical outcomes including mood disturbance, impairment in cognitive functioning, metabolic dysfunction, and osteopenia.

## Ageing, DHEA and DHEAS

**Fig. 2:**
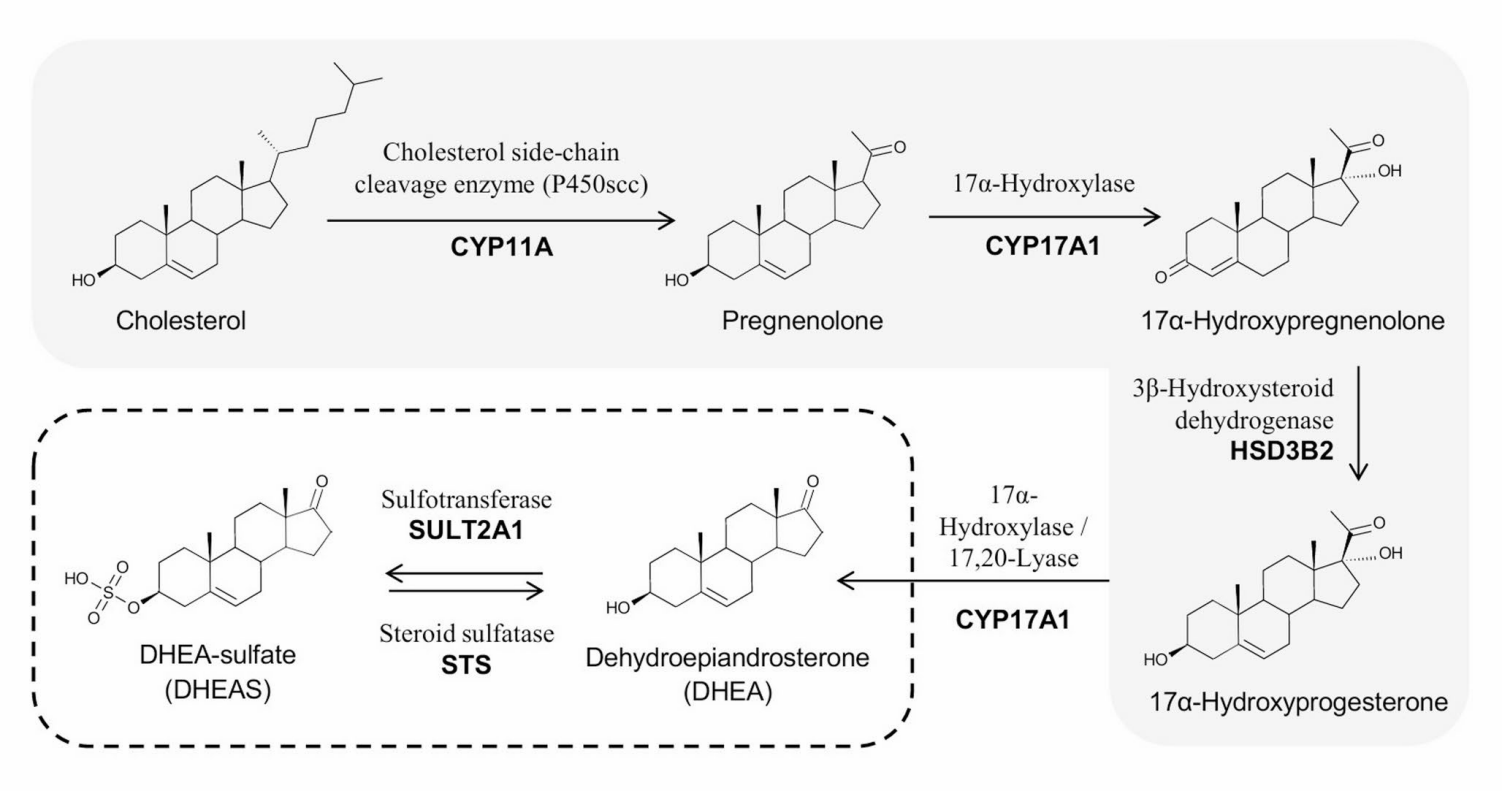
Biosynthetic pathway of DHEA/ DHEAS

DHEA is the most abundant steroid hormone in primates [6, 7]. It is produced from cholesterol, largely in the adrenal glands, but also in the testis, ovaries, skin, and brain [7]. DHEAS is produced from DHEA in the ZR (Fig. 2) [7]. Both DHEA and DHEAS are secreted by the adrenal glands in response to adrenocorticotropic hormone (ACTH), with DHEAS loosely bound to plasma albumin, acting as a DHEA reserve. DHEAS is converted in tissues by sulfotransferases and hydroxysteroid sulfatases back to DHEA, which is the physiologically active steroid (Fig. 2) [7].

The adrenal glands produce large amounts of DHEA and DHEAS during foetal development, but production falls rapidly after birth and remains low during the first five years of life [6, 8, 9]. Subsequently, levels rise and peak during the second to third decades, during the ‘adrenarche’ [6, 9]. Circulating levels of DHEAS are higher than that of DHEA, and levels are both age and sex dependent, with higher levels in males than females [8]. After the third decade, there is an age-dependent decline in circulating DHEA and DHEAS [9, 10], such that by the seventh decade of life, DHEAS levels may have dropped to 10–20% of their maximum concentration [6, 10].

Serum DHEAS concentration remains stable throughout the day, whereas secretion of DHEA follows a diurnal rhythm, similar to that of cortisol [6]. In addition to reduced circulating levels of DHEA with increasing age, an attenuation of the diurnal rhythm and the pulse amplitude of DHEA secretion has also been described [6].

### Functions of DHEA

DHEA is a crucial sex steroid precursor [6, 8]. It is converted to androstenedione by the activity of 3β-hydroxysteroid dehydrogenase (3β-HSD) and then further converted to testosterone and oestradiol by isoenzymes of 17β-HSD and P450 aromatase, respectively [6]. DHEA is also converted to intermediate steroids that may have distinct activity, for example androstenediol [6]. In post-menopausal women, ovarian production of oestrogens and DHEA falls to almost zero, making adrenals the main source of oestrogens and testosterone through DHEA [7].

In addition to their role in the production of sex hormones, DHEA and DHEAS are proposed to have effects in the central nervous system and on the immune system [8]. Both DHEA and DHEAS are neuroactive steroids in the brain, directly interacting with sigma, glutamate, *N*-methyl-d-aspartate (NMDA) and γ-aminobutyric acid (GABA_A_) receptors [6, 8], through which they have been hypothesised to exert anti-depressant and anxiolytic effects [6]. Using animal and in vitro studies, it has been shown that DHEAS stimulates neuronal growth and development and improves glial survival, as well as modulating cognitive functions such as learning and memory [6].

DHEA has differential actions on human immune function, and its effects are impacted by concentrations of other hormones [11]. DHEA may have anti-glucocorticoid activity and may modulate inflammation and cytokine responses to stimulation in a variety of contexts [11].

### Ageing-related DHEA deficiency and clinical outcomes

Low serum levels of DHEAS that occur with ageing are associated with multiple adverse clinical outcomes, including an increased risk of all-cause mortality and cardiovascular disease mortality in males, and poor functional status in males [6, 12]. Low DHEAS concentrations have been reported in systemic lupus erythematosus (SLE), dementia, breast cancer and rheumatoid arthritis, and generally there is an inverse relationship between serum DHEAS levels and severity of disease [6]. DHEA deficiency is also associated with several major neurodevelopmental and neurodegenerative pathologies, including schizophrenia, bipolar affective disorder, depression and Alzheimer’s disease [8], diabetes [13] and low bone mineral density [14].

However, association does not equate to causation, and further research is required to determine whether low circulating DHEA/DHEAS is contributing to functional decline or whether it is a physiological component of ageing. It is feasible that low circulating DHEA/DHEAS is merely associated with end organ pathology, or it may be a consequence of a disease process, as chronic disease can lead to a shift in intra-adrenal biosynthesis away from DHEA/DHEAS production and towards cortisol secretion [6].

Clinical trials that have been carried out in individuals considered to be healthy, older adults to determine whether oral DHEA may have an effect on parameters such as well-being, mood, cognition, sexual function and activities of daily living have largely reported that DHEA does not have a significant beneficial therapeutic effect [15–18]. While previous work documented a possible improvement in physical and psychological well-being following DHEA supplementation, a meta-analysis of eight studies (*n* = 661 participants) failed to demonstrate a conclusive effect for DHEA on muscle strength and physical function in older adults [19]. Studies on bone health with DHEA supplementation have shown a possible benefit in older women but no effect was observed in men [20]. However, these findings were far from conclusive due to the limited number of study participants and the heterogeneity across studies. Others have shown improvement in carotid augmentation index, suggesting a potential benefit on vascular health [21] but hard vascular outcome studies are lacking. Overall, studies on DHEA supplementation are, on the whole, inconclusive, which can be attributed to different factors. First, the number of participants in the various studies to date tended to be small and therefore they were not adequately powered to demonstrate an effect. Second, studies were conducted over a short period of time, some as short as weeks, and therefore it is unclear whether an extended treatment would have had an effect. Third, many studies focused on subjective end points, such as measures of well-being, and more work is needed to investigate hard clinical outcomes. Finally, it is possible that a lack of efficacy could be related to selection bias, as generally only seemingly healthy subjects with excellent performance status at baseline were included in the studies.

### Pathophysiology of reduced secretion of DHEA/ DHEAS

Adrenal glands in older individuals have a smaller ZR than in younger individuals, which could equate to a reduced number of DHEA-secreting cells [6, 9, 22]. Furthermore, research in non-human primates (NHP) has demonstrated that there are ageing-associated transcriptional changes in the cells of the ZR, which has a major impact on lipid metabolism [1]. The low-density lipoprotein receptor gene (*LDLR*) is one of the prominently downregulated genes [1]. The lipoprotein receptor is vital in internalising lipoprotein particles and is a carrier for cholesterol. In cultured human cells, *LDLR* knockdown results in reduced intracellular cholesterol, which is the precursor for steroid hormones, and decreased secretion of DHEAS [1].

*SULT2A1* is another example of a downregulated gene in the ZR. It encodes the steroid sulfotransferase that catalyses DHEA sulfation [1] so its downregulation could be another factor contributing to reduced secretion of DHEAS [1].

### Ageing and aldosterone

The trajectory of aldosterone secretion with increasing age remains controversial, with some evidence of an ageing-related decline [23] and some evidence of greater autonomous aldosterone secretion with ageing [24].

Aldosterone production is primarily regulated by angiotensin (Ang) II, circulating potassium levels and ACTH [24]. The renin-angiotensin-aldosterone system (RAAS) contributes towards the homeostatic regulation of blood pressure and serum sodium concentration [25]. The RAAS is under feedback control via the release of renin from the renal juxtaglomerular cells, that is in turn influenced by numerous factors including blood pressure, renal perfusion pressure and hyponatraemia [25].

**Fig. 3:**
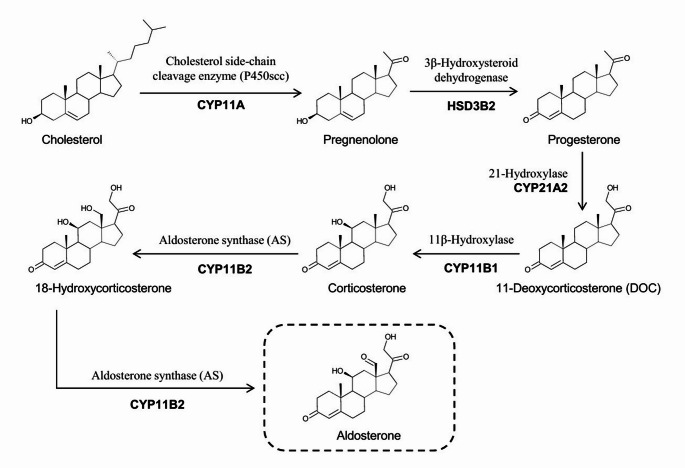
Biosynthetic pathway of aldosterone

Aldosterone is the primary mineralocorticoid and is synthesised within the ZG of the adrenal cortex [24]. Aldosterone acts within the renal tubule to increase sodium retention, thereby maintaining intravascular volume and blood pressure [24]. The synthesis of aldosterone within the ZG is regulated by Ang II and serum potassium concentration [24]. It generated from cholesterol through a series of enzymatic reactions that include cholesterol side-chain cleavage (CYP11A1), type 2 3β-hydroxysteroid dehydrogenase (HSD3B2), 21-hydroxylase (CYP21A2), and CYP11B2 [24] (Fig. 3). CYP11B2 is expressed specifically in the ZG such that aldosterone production is confined to the ZG [24].

In contrast to DHEA and DHEAS, relatively little is known about the ageing-related changes in aldosterone synthesis/secretion from the adrenal cortex [24]. However, there is a correlation between age and RAAS activity in humans, with levels of plasma renin activity and plasma aldosterone being highest in the newborn, and lowest in the elderly population [26–28] (Table 1). RAAS function is also influenced by race and sex [26].

**Table 1:** The correlation between Renin and aldosterone levels and chronological age

	≤35 years old	≥62 years old	*P* Value	References
Renin (ng/ml^a^)	41.1 ± 4.1	26.4 ± 5.7	< 0.05	[27]
Aldosterone (ng/dl^b^)	12.6 ± 2.6	5.6 ± 0.8	< 0.05	
Aldosterone reduction per year (ng/dl)	0.18–0.25	ND	*	[28]

^a^Millilitre; ^b^Decilitre; ND = not determined

Older age appears to be associated with a blunted ability to secrete aldosterone in response to its regulators, Ang II and potassium [24]. The reductions of plasma renin activity and plasma aldosterone levels with increasing age are usually modest, and do not usually associate with changes in fluid and electrolyte homeostasis [26]. However, suboptimal functioning of the RAAS in older people can result in increased vulnerability to any concurrent compromise to haemodynamic function or additional diminishment of the RAAS [26]. This can result from, for example, heart failure, or the use of Angiotensin-Receptor Blocking (ARB) or Angiotensin-Converting Enzyme (ACE) inhibitor therapies, respectively. In addition to the clinical implications of an ageing-related decline in RAAS function, such ageing-related changes in the RAAS have implications for the accurate diagnosis of RAAS dysfunction. Although not commonly used within lab-based reporting, there is an argument for age-related normal ranges for plasma renin activity and plasma aldosterone levels to assist clinical decision-making in relation to the correction of age-related RAAS dysfunction.

**Fig. 4:**
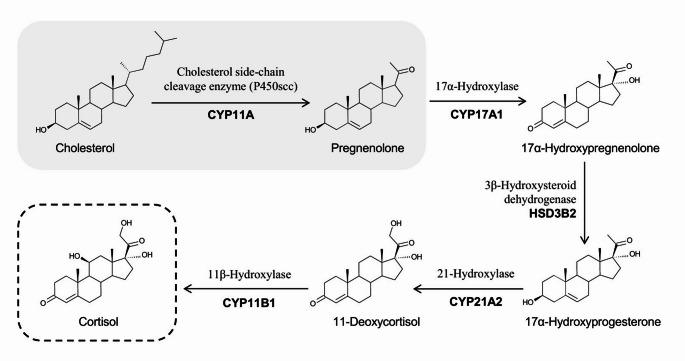
Biosynthetic pathway of cortisol

To complicate the picture of serum aldosterone levels with ageing, there are ageing-related histological changes in the expression of adrenal CYP11B2, the enzyme responsible for aldosterone synthesis within the ZG [24]. Indeed, non-neoplastic foci of aldosterone-producing cell clusters (APCC) that express CYP11B2 are a common occurrence in normal human adrenals, and histopathological studies show an association between older age and greater adrenal APCC content [29–31] with progressive autonomous aldosteronism. Furthermore, there is an ageing-related decline in CYP11B2 expression in the ZG [24], whilst transcriptome analysis of APCC has demonstrated that APCC messenger RNA (mRNA) profiles have similar characteristics to those of ZG but with higher *CYP11B2* expression, indicating an increased capacity to produce aldosterone [29].

Therefore, with advancing age there seems to be a transition from continuous expression of CYP11B2 within the ZG to APCC predominance [24, 29], and a concomitant migration from normal physiological aldosterone regulation through the RAAS towards autonomous and renin-independent aldosterone secretion [24]. This scenario may underlie some of the age-related increase in hypertension and risk for CV disease [24]. However, most research on human APCC has been carried out either on postmortem adrenal glands or adrenal glands containing lesions, therefore the biochemical phenotype of APCC has not been specifically quantified [29].

In summary, with increasing age, there seems to be a juxtaposition between a general decline in RAAS functioning, that can manifest in a general reduction in serum aldosterone levels and vulnerability to hypotension and cardiovascular compromise, and concurrently an ageing-associated dissociation of aldosterone synthesis from RAAS control to autonomy that can also result in hypertension and contribute to cardiovascular disease. Interestingly, the impact of aberrant aldosterone levels is not just confined to the cardiovascular and urinary systems. Presbyacusis is associated with reduced aldosterone levels, suggesting that aldosterone may have a protective effect on hearing [32]. Aldosterone plays a role in the maintenance of key ion pumps, including the Na-K-Cl co-transporter 1 or NKCC1, which is involved in homeostatic maintenance of the endocochlear potential [33].

### Ageing and cortisol

Glucocorticoids are steroid hormones produced mainly in the ZF, with cortisol being the most potent and responsible for 95% of all glucocorticoid activity [34]. In response to stress, the hypothalamus secretes corticotropin-releasing hormone (CRH), which enters the hypothalamic-hypophysial portal circulation and stimulates the anterior pituitary gland to release ACTH [30]. ACTH then triggers the adrenal gland’s secretion of adrenocortical hormones [34]. Cortisol exhibits a pulsatile and diurnal secretion pattern, with the highest concentration secreted in the morning and the lowest in the evening [34]. Cortisol exhibits negative feedback on both the hypothalamus and the pituitary gland, and has multiple physiological actions, including stimulating gluconeogenesis and glycogen storage, inducing lipolysis and proteolysis, an anti-inflammatory role and anti-osteoblastic effects [34].

Cortisol secretion appears to increase with advancing age, with mean cortisol levels increasing by 20–50% between the ages of 20 and 80 years in both males and females [35]. Premenopausal women have slightly lower mean levels than men in the same age range, primarily because of lower morning maxima [35]. Furthermore, whilst the diurnal rhythmicity of cortisol secretion is preserved with increasing age, the relative amplitude is dampened, and the timing of the circadian elevation is advanced [35].

In one of the most robust studies that addresses how cortisol levels change with ageing, Moffat et al. measured 24-hour urinary free cortisol (UFC) and creatinine (Cr) in 1,814 individuals from the Baltimore Longitudinal Study of Ageing for a follow-up period of up to 31 years [36]. The pattern and slope of cortisol levels were assessed from ages 20 to 90 years and older [36]. UFC/Cr followed a U-shaped pattern across the life span, with decreases in UFC/Cr in the 20s and 30s, relative stability in the 40s and 50s, and increases thereafter [36].

It has also been shown that with ageing, reduced sensitivity to glucocorticoid feedback signals is acquired [37]. Forty men, with a mean age of 69 +/- 5 years, and 20 younger individuals, with a mean age of 34 +/- 8 years underwent a combined dexamethasone suppression/CRH-stimulation test. The study participants were pre-treated with dexamethasone (DEX) and were then administered CRH. Following this intervention, the older men released significantly more cortisol than the younger cohort, and in the older group only, there was a positive correlation between basal, DEX-pretreated cortisol concentration and post-CRH steroid responses [37].

The mechanisms for increased cortisol secretion need to be further evaluated. As described above, it has been proposed that with ageing there is reduced negative feedback to endogenous corticosteroid levels [36, 37], and animal models have demonstrated reduced number of glucocorticoid receptors in the hippocampus, prefrontal cortex, and hypothalamus [38]. Furthermore, proinflammatory cytokines that are secreted in a range of ageing-associated metabolic, somatic, and psychiatric conditions may act on multiple levels of the hypothalamic–pituitary–adrenal system ultimately increasing glucocorticoid secretion [36].

Increased cortisol with ageing is associated with impairment in some aspects of cognitive functioning, such as tasks measuring explicit memory and selective attention [39]. This is proposed to be driven by an increased cortisol: DHEA ratio, with chronically high cortisol levels exerting a catabolic neurotoxic effect, leading to reduced dendrite length and neuronal death [40]. Furthermore, higher cortisol levels are significantly associated with smaller left hippocampal volumes and are negatively correlated with memory function through hippocampal volume [41]. Higher cortisol levels are also associated with lower grey matter volume in the temporal and parietal areas in the left hemisphere [40].

It is also feasible that raised cortisol could have a causative role in increasing the risk of diseases such as diabetes, obesity, hypertension, osteoporosis and cardiovascular disease [42]. For example, a prospective cohort study in a non-clinical population of over 60s found that disturbances in diurnal cortisol secretion, as well as raised evening cortisol levels, were associated with type II diabetes onset [43]. However, research exploring the association of cortisol levels and body mass index (BMI) found no correlation [44], suggesting that the role of cortisol in metabolic syndrome is still unclear. It has also been shown that patients with autonomous cortisol secretion, due to adrenal hyperplasia or the presence of an adenoma, exhibit reduced suppression of post-dexamethasone suppression test (DST) cortisol, 11-deoxycortisol, and corticosterone, with post-DST cortisol and corticosterone being associated with a higher prevalence of severe/resistant hypertension [45]. It is also plausible that ageing-related hypercortisolism influences immune dysfunction, potentially increasing the risk of infections [46].

### Structural and functional changes of the adrenal glands with increasing age

Animal models have helped to progress our understanding of the structural and functional changes of the adrenal gland that occur with ageing. Research in non-human primates (NHP) demonstrated an accumulation of p21^Cip1^-positive cells in the ZG, ZF, ZR and medulla, along with deposition of aggresome, which is regarded as a marker of tissue ageing [2]. Furthermore, it has been shown that there is an abnormal accumulation of amyloid-β peptide, in the ZR, ZG and medulla of aged adrenal glands, along with increased expression of GPNMB, a seno-antigen expressed by senescent cells, in ZR, ZG, ZF and medulla. Abnormal deposition of lipofuscin is also identified in the ZR [2].

Other observations in aged NHP adrenal glands are that there is impaired cortical differentiation, resulting in impaired formation of the ZR, and there are increased numbers of T cells and macrophages. This is associated with dysregulation of cell-cell communication and may contribute to enhanced inflammatory responses [2]. Transcriptome analysis has shown that genes involved in hormone metabolism are downregulated, whereas genes associated with cytokine production and leukocyte cell–cell adhesion are activated in aged adrenal tissues [2].

With increasing age, there is an increase in the prevalence of adrenal tumours. It has been reported that more than 90% of adrenal tumours are found in patients older than 40 years of age, with a median age at diagnosis of 62 years (*n* = 1287) [47].

Thus far, research into the structural changes of the ageing adrenal glands have largely been carried out using animal models. There is a paucity of data from human studies, therefore future research in humans should be a research priority.

### Is adrenal cortex senescence an ageing-related pathology?

Adrenal cortex senescence is an emerging entity which appears to fulfil the criteria for an ageing-related pathology:

1. Functional changes are observed with increasing chronological age, in particular there is reduced secretion of DHEA and DHEAS, and there is increased output of cortisol.
2. Such changes are associated with a range of adverse clinical outcomes, including an increased risk of premature mortality, SLE, dementia, breast cancer, rheumatoid arthritis, schizophrenia, bipolar affective disorder, depression, Alzheimer’s disease, diabetes and low bone mineral density.
3. These findings have been reported in studies carried out in humans.

However, further evidence is required before adrenal cortex senescence can be definitively regarded as such. Whilst numerous diseases are associated with low serum DHEA/DHEAS, this may just be an association, or a consequence of the disease process. It remains to be determined whether reduced secretion of DHEA/DHEAS has any pathological outcomes.

Similarly, it is important to advance the understanding of whether the increased cortisol output observed with increasing age mediates any adverse clinical effects, its underlying pathophysiology, and to better characterise the ageing-related changes in aldosterone secretion.

Furthermore, much of the research considering the structural and morphological changes of the ageing adrenal gland has been carried out in animal models, and evidence from human studies is relatively scarce.

## Summary

Our hypothesis is that structural and functional changes of the adrenal cortex develop and progress with increasing age, resulting in reduced secretion of DHEA/DHEAS and increased secretion of cortisol. It is important to obtain further evidence to better characterise the degenerative changes of the adrenal cortex, and to elucidate the clinical consequences of this.

If adrenal cortex senescence is to be considered as an ageing-related pathology, methods for its diagnosis and staging would need to be determined. This could then lead to opportunities to develop interventions to halt, reverse, or slow its progression, to improve the quality of life of individuals and to promote healthy longevity.

On behalf of all authors, the corresponding author states that there is no conflict of interest.
